# Are growing inequities leaving Africa behind in the post-pandemic public health landscape?

**DOI:** 10.11604/pamj.2024.47.16.41653

**Published:** 2024-01-15

**Authors:** Simon David Taylor-Robinson, Andrew William Taylor-Robinson

**Affiliations:** 1 Department of Medicine, Busitema University, Mbale, Uganda,; 2 Department of Public Health, Busitema University, Mbale, Uganda,; 3 Faculty of Engineering, Imperial College London, London, United Kingdom,; 4 College of Health Sciences, VinUniversity, Hanoi, Vietnam,; 5 Center for Global Health, Perelman School of Medicine, University of Pennsylvania, Philadelphia, USA

**Keywords:** COVID-19, pandemic, Africa, public health, inequity

## To the editors of the Pan African Medical Journal

Coronavirus disease 2019 (COVID-19) has exerted significant economic and social impacts globally, Africa bearing a major burden [1]. These community-wide effects have been more severe compared to previous pandemics, such as the Spanish Flu and Ebola outbreaks. Studies have examined various aspects, including finance, stock market volatility, sustainable agriculture, and income inequality, to assess the effects of the pandemic on Africa [2,3]. The economic consequences have been substantial, with disruptions to financial markets and institutions [2]. In addition, the pandemic has highlighted existing challenges in healthcare infrastructure and public healthcare services across the continent [1,4]. Africa's socioeconomically disadvantaged populations have been disproportionately affected by the pandemic, exacerbating such issues as poverty, undernourishment, and limited access to healthcare [1,4]. National healthcare systems have faced significant strain, exacerbating the existing need for more efficient and prepared public healthcare services [3]. Notably, the pandemic has worsened income-related health inequities and gender disparities [5]. The combination of stay-at-home orders, lockdowns, and shutdowns has had a tremendous impact on vulnerable populations during the pandemic and in the period immediately after easing of restrictions, thereby worsening deep-seated structural and societal inequalities [5]. Moreover, the pandemic has highlighted the need for sustainable urban growth strategies and equitable development in regional towns and cities in all countries [6].

In a post-COVID-19 pandemic world, concerns have been raised about whether Africa's public health needs are being left behind. The emergence of severe acute respiratory syndrome coronavirus 2 (SARS-COV-2) has exacerbated existing challenges in healthcare infrastructure, funding stability, healthcare provider knowledge, treatment availability, and disease registries in Africa [1,3]. Moreover, studies have highlighted the strain on healthcare systems, the decline in public health services, and the mental health challenges faced by healthcare professionals in Africa during the pandemic [3,7]. Furthermore, the pandemic has deepened regional disparities in access to primary healthcare, potentially leading to inequalities in health outcomes [1]. These inadequacies had already resulted in a substantial drop-off of patients along the care cascade, with many going undiagnosed or not receiving the necessary advice and treatment [1,7,8]. For instance, the cost of diabetes and its complications in sub-Saharan Africa is considered to be significant, with both direct payments and out-of-pocket expenditures placing a burden on individuals and economies [8].

Limited clinical data and research on public health priorities, such as dementia prevalence, further hinder progress in addressing long-established health needs [9]. In order to address these challenges and prioritize public health needs in Africa, several actions can be taken. First, there is a need for comprehensive data collection to understand the burden of diseases and health disparities in the region [4,9]. This will help raise awareness and generate political commitment to make public health a priority. Additionally, investment in healthcare infrastructure, sustainable funding, and emergency preparedness is crucial to build resilience and mitigate the impact of future public health events [2,6]. Efforts should also focus on strengthening primary healthcare systems, ensuring equitable access to healthcare, and addressing socioeconomic disparities. Furthermore, interventions that connect individuals to healthcare resources and promote health-promoting behaviors should be implemented following economic migration or during societal transitions, such as from jail to the community [2].

Another example is consultation with rural community leaders to incorporate local strategies for livestock rearing and prevent contamination of water bodies [2]. Allied with this, investment in water, sanitation, and hygiene (WASH) facilities, along with effective habitat modification strategies, is crucial for controlling water-borne diseases like schistosomiasis [10]. Reversing growing public health inequities in the aftermath of COVID-19 requires a multi-faceted approach that includes policy changes, resource allocation, and efforts to reduce structural inequalities. It is crucial to prioritize health equity, improve job security and work-life balance for healthcare professionals, and promote diversity and inclusion in academic and management positions. By addressing these priorities, Africa can work towards equitable development and ensuring that public health needs are not left behind by the rest of the world in the post-pandemic era.
